# Reply to Zhou et al.: Exercise, caloric restriction, and immunotherapy responsiveness in 4T1 breast cancer progression

**DOI:** 10.1073/pnas.2617811123

**Published:** 2026-07-17

**Authors:** Amanda Garrido, Nathan L. Price, Michel Bernier, María Carmen Gómez-Cabrera, Rafael de Cabo

**Affiliations:** ^a^ Experimental Gerontology Section, Translational Gerontology Branch, National Institute on Aging, NIH, Baltimore, MD 21224; ^b^ Freshage Research Group, Department of Physiology, Faculty of Medicine, University of Valencia, Centro de Investigación Biomédica en Red Fragilidad y Envejecimiento Saludable, Fundación para la Investigación del Hospital Clínico de la Comunidad Valenciana, Valencia 46010, Spain

In a Letter to the Editor in response to our recent PNAS publication, Zhou et al. propose a possible explanation for our findings regarding the effects of exercise on tumor progression (1, 2). They emphasize the importance of the tumor microenvironment (TME) and hypothesize that response to immunotherapy may be closely linked to the host’s capacity to benefit from exercise. We agree that the behavior of the different cell types within the TME, as well as the status of the immune system, play critical roles in how tumor cells respond to treatment, promote angiogenesis, and drive other tumor-related processes relevant to metastasis. Importantly, our study did not include immunotherapy or a direct assessment of responsiveness to immune checkpoint blockade; therefore, the proposed mechanism remains speculative in the context of our findings.

The 4T1 mouse model of breast cancer used in our study is widely considered nonimmunogenic, immunosuppressive, and resistant to checkpoint blockade monotherapies (3, 4). However, it is not entirely unresponsive to combination therapy, and other groups have reported positive responses to various immunotherapeutic approaches (5, 6). Previous work from our laboratory has characterized the effects of caloric restriction (CR) on the immune profile of this model and the implications for tumor growth and metastasis (7, 8). CR reduces the frequency of immunosuppressive cell populations, such as regulatory T cells and myeloid-derived suppressor cells (MDSCs), while increasing CD4^+^ and CD8^+^ T cells, resulting in smaller tumors and reduced metastatic spread. When MDSCs are inoculated into CR mice bearing 4T1 tumors, the primary tumors are no longer smaller than those in ad libitum-fed (AL) mice, highlighting the key role of CR-mediated reduction in MDSCs in inhibiting tumor growth. However, metastasis remains decreased in these mice, suggesting that the effects of CR on tumor cell migration into the bloodstream and/or engraftment in the lungs are independent of this mechanism.

In the context of the hypothesis presented by Zhou et al., CR mice would be expected to derive greater benefit from exercise than AL mice because their immune systems are more competent and less exhausted. Additionally, if the immune response were the key factor determining whether exercise promotes or suppresses cancer progression, we would expect a consistent response across both primary tumor growth and metastasis. However, we observed a detrimental effect only on metastasis, with no differences in primary tumor growth. The inclusion of chemotherapy across all groups in our study is an important variable to consider, as we do not know how it may have influenced the previously described effects of CR on the immune response. Furthermore, the increased robustness of the immune response observed in CR mice may not be sufficient to produce a beneficial response to exercise.

While we believe the current literature, our previous work, and the study that inspired this Letter do not fully support the ideas put forward by Zhou et al., we appreciate the insights and discussion they offer and agree that their hypothesis warrants further investigation.

## Data Availability

There are no data underlying this work.
